# Copper-Doped Ordered Mesoporous Bioactive Glass: A Promising Multifunctional Platform for Bone Tissue Engineering †

**DOI:** 10.3390/bioengineering7020045

**Published:** 2020-05-21

**Authors:** Francesco Baino

**Affiliations:** Institute of Materials Physics and Engineering, Department of Applied Science and Technology, Politecnico di Torino, 10129 Turin, Italy; francesco.baino@polito.it

**Keywords:** biomaterials, bioglass, porosity, bioactivity, antibacterial, tissue engineering

## Abstract

The design and development of biomaterials with multifunctional properties is highly attractive in the context of bone tissue engineering due to the potential of providing multiple therapies and, thus, better treatment of diseases. In order to tackle this challenge, copper-doped silicate mesoporous bioactive glasses (MBGs) were synthesized via a sol-gel route coupled with an evaporation-induced self-assembly process by using a non-ionic block co-polymer as a structure directing agent. The structure and textural properties of calcined materials were investigated by X-ray powder diffraction, scanning-transmission electron microscopy and nitrogen adsorption-desorption measurements. In vitro bioactivity was assessed by immersion tests in simulated body fluid (SBF). Preliminary antibacterial tests using *Staphylococcus aureus* were also carried out. Copper-doped glasses revealed an ordered arrangement of mesopores (diameter around 5 nm) and exhibited apatite-forming ability in SBF along with promising antibacterial properties. These results suggest the potential suitability of copper-doped MBG powder for use as a multifunctional biomaterial to promote bone regeneration (bioactivity) and prevent/combat microbial infection at the implantation site, thereby promoting tissue healing.

## 1. Introduction

Over the last years, there has been an increasing interest in investigating the biological effects that can be elicited by ionic dissolution products released by implanted biomaterials. In fact, it is known that several trace elements are involved in cell metabolic processes and act as enzyme cofactors, thereby playing key roles in regulating many functions of the body [1]. Hence, the controlled release of dopants from biomaterials is a valuable approach to modulate the therapeutic response and, ultimately, promote healing and regeneration in tissue engineering strategies [2,3].

Bacterial infection is one of the major causes hindering tissue healing and leading to implant failure; in this regard, a special set of metallic cations with antimicrobial properties (e.g., Ag^+^, Ga^3+^, Cu^2+^) has been suggested for therapeutic purposes. Silver has been well-known to have a bactericidal activity since ancient times [4]. Silver ions are more effective against Gram-negative bacteria than Gram-positive species. The antibacterial effect of Ag^+^ ions is associated to the silver affinity with disulfide (S–S) and sulfhydryl (–SH) groups available on the proteins of microbial cell walls. As a result of the binding reaction with silver, normal metabolic processes of bacteria, such as oxidative metabolism and uptake of nutrients, are disrupted leading to cell death [5].

The antibacterial properties of gallium are due to the competition that Ga^3+^ ions establish with Fe^3+^ ions in many biochemical reactions owing to the similarity of their ionic radii (“Trojan horse” effect). Uptake of Ga^3+^ ions leads to the inhibition of some key biological reactions in bacteria, such as those involved in DNA and protein synthesis [6]. 

Copper ions can kill bacteria due to the generation of reactive oxygen species (ROS), lipid peroxidation, protein oxidation and DNA degradation [7]. Copper ions exhibit good antibacterial activity against both Gram-positive and Gram-negative bacteria [8] and, very interestingly, can stimulate the formation of collagen by bone cells, thereby contributing to osteogenesis and inhibiting osteoporosis [9]. All these attractive features make copper a valuable dopant to be incorporated in bioactive ceramics and glasses for making multifunctional biomaterials, which combine osteoconduction/osteoinduction with new therapeutic extra-functionalities.

Copper-doped hydroxyapatite microspheres have been prepared by chemical co-precipitation [10], high-temperature solid-phase synthesis [11], ion-exchange methods [12] and pneumatic extrusion printing [13].

Incorporation of copper in bioactive silicate glasses has been reported via melt-quenching route [14] or sol-gel process [15]. Surface functionalization of sol-gel glasses with copper nanoparticles was achieved by applying impregnation routes and proper thermal treatments [16]. Resorbable copper-doped phosphate glass fibers were also fabricated by drawing for potential application in wound healing and skin tissue engineering [17]. Doping of sol-gel silicate glass compositions with copper has been recently proposed as an interesting approach for obtaining multifunctional biomaterials combining tissue regenerative and antibacterial capabilities [18,19,20,21]. Specifically, the use of ion-doped biomedical glasses in the context of antibiotic-free antibacterial applications has been reviewed by Kaya et al. [22].

This work reports the synthesis of copper-doped glasses via a modified sol-gel method incorporating supramolecular chemistry, which allows mesoporous bioactive materials to be obtained.

## 2. Materials and Methods

### 2.1. Preparation

The process used for the synthesis of copper-doped silicate glasses was a sol-gel-type route commonly known as the evaporation-induced self-assembly (EISA) method, which is applied to produce mesoporous materials. The parent binary glass belonged to the 80SiO_2_-20CaO (mol.%) system; CuO was introduced to partially substitute CaO in the glass composition, thus obtaining 80SiO_2_-19CaO-1CuO (1Cu-glass) and 80SiO_2_-15CaO-5CuO (5Cu-glass) formulations (mol.%).

The glass synthesis procedure was adapted from that reported by Yan et al. [23] for the preparation of mesoporous silicate glasses, which initially did not contain copper. The non-ionic block copolymer EO_20_-PO_70_-EO_20_ (Pluronic P123, M_w_ = 5800 g/mol, Sigma-Aldrich, St. Louis, MO, USA) was used as a structure-directing agent, while tetraethoxysilane (TEOS), calcium nitrate tetrahydrate (Ca(NO_3_)_2_·4H_2_O) and copper chloride (CuCl_2_) (all the reagents were purchased from Sigma-Aldrich, St. Louis, MO, USA) were used to supply SiO_2_, CaO and CuO, respectively. Firstly, 4.0 g of Pluronic P123 were dissolved in 60.0 g of ethanol with 1.0 g of 0.5 M HCl used as a catalyst under constant stirring at room temperature; then, once Pluronic P123 was completely dissolved, TEOS and salts were slowly added over 3 h following this order: 6.7 g of TEOS, 1.8 or 1.425 g of Ca(NO_3_)_2_·4H_2_O (for 1Cu-glass and 5Cu-glass, respectively), and 0.054 or 0.27 g of CuCl_2_ (for 1Cu-glass and 5Cu-glass, respectively). The sols were then poured into Petri dishes to allow the EISA process to occur at room temperature. Aged gels were removed from the dishes and calcined in air at 650 °C for 5 h (heating and cooling rate of 2 and 5 °C/min); the selection of calcination temperature was also performed according to the results from thermogravimetric analysis (TGA) on the gels. The calcined materials were finally ground by ball milling (Pulverisette 0, Fritsch, Idar-Oberstein, Germany) and sieved by stainless steel sieves with a mesh of 32 μm (Giuliani Technologies, Torino, Italy). 

### 2.2. Characterization

Calcined materials underwent wide-angle (2θ within 10–60°) X-ray powder diffraction (XRPD) by using a X’Pert Pro PW3040/60 diffractometer (PANalytical, Eindhoven, The Netherlands) operating at 40 kV and 30 mA with Bragg-Brentano camera geometry and Cu Kα incident radiation (wavelength λ = 0.15405 nm) in order to assess the presence of crystalline phases. 

Small-angle XRPD (2θ within 0.8–4°) was also performed in order to assess the presence of an ordered pore symmetry in the materials.

Textural parameters were assessed by nitrogen adsorption-desorption measurements performed at −196 °C (Quantachrome Autosorb1, Quantachrome, Boynton Beach, FL, USA). The Brunauer-Emmet-Teller (BET) method [24] and the density functional theory (DFT) isotherm reconstruction approach [25] were used to determine pore volume, specific surface area (SSA), pore size distribution and mean pore size.

The porous structure was also examined by means of scanning-transmission electron microscopy (STEM) (Merlin, Zeiss, Oberkochen, Germany) operating at 30 kV.

In vitro bioactivity was assessed in terms of apatite-forming ability by immersion tests in simulated body fluid (SBF) for 2 weeks. SBF was prepared according to the protocol recommended by Kokubo and Takadama [26]. Small cylinders of pressed powders (diameter = 10 mm, height = 5 mm) were maintained in polyethylene bottles filled with SBF at 37 °C in a static incubator; a ratio of sample mass to solution volume of 1.5 mg/mL was used, as recommended in previous studies [27,28]. At the end of the experiment, the samples were extracted, rinsed with ethanol to stop reactions, left to dry overnight at room temperature and analyzed by SEM equipped with energy-dispersive spectroscopy (EDS) probe (SEM-EDS, Merlin, Zeiss, Oberkochen, Germany) after being sputter-coated with chromium. The sample surface was also analyzed by X-ray diffraction (XRD) method according to the procedure described above.

Antibacterial activity was assessed against a standard *Staphylococcus aureus* strain by performing the Kirby-Bauer test according to the Performance Standards for Antimicrobial Disk Susceptibility Test (Approved Standard, 9th Ed., NCCLS, Villanova, PA, USA, 2006).

## 3. Results and Discussion

**Figure 1:**
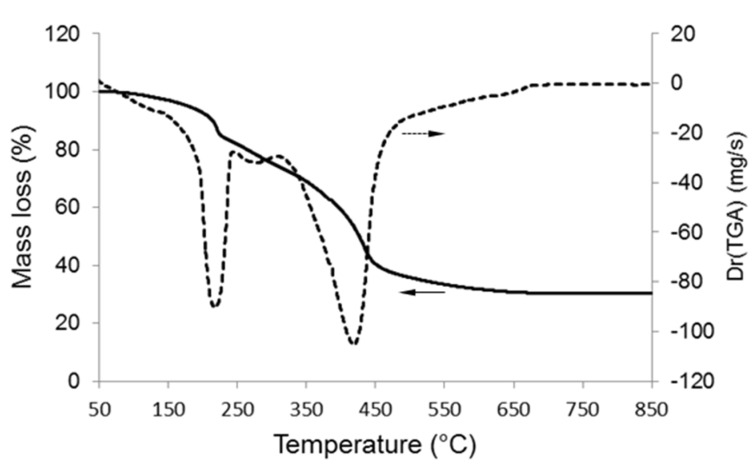
Thermogravimetric analysis (TGA) of the gel corresponding to the 80SiO_2_-20CaO (mol.%) nominal composition: mass loss and lass loss derivative.

TGA was useful to select the calcination temperature (Figure 1). An increase of mass loss up to 650 °C was observed (about 30% of total mass loss); above this temperature, the mass remained constant, confirming that the surfactant was completely removed from the material. Mass reduction could be attributed to two major events associated to the removal of ethanol and water from the gel (70–200 °C) and the thermal decomposition of organics (surfactant) and nitrates (300–500 °C).

**Figure 2:**
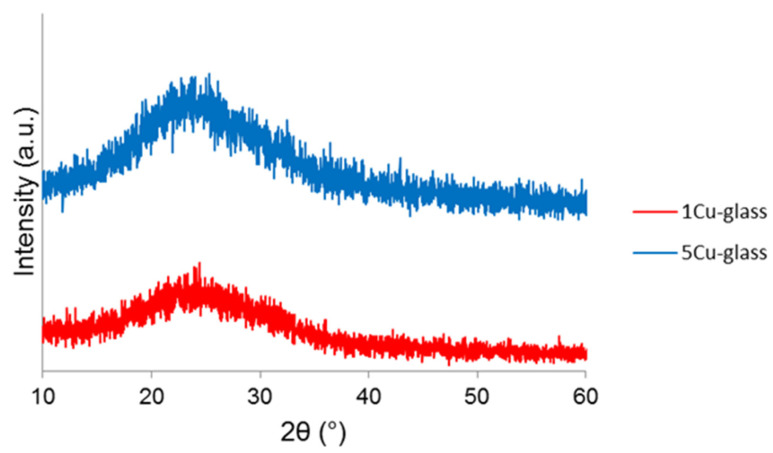
Wide-angle X-ray powder diffraction (XRPD) patterns of copper-doped materials after calcination.

The wide-angle XRPD patterns of calcined 1Cu-glass and 5Cu-glass are displayed in Figure 2 and confirm the amorphous nature of both materials, as proved by the presence of only broad haloes in the 2θ range of 15 to 35°, which is typical of silicate glasses. 

**Figure 3:**
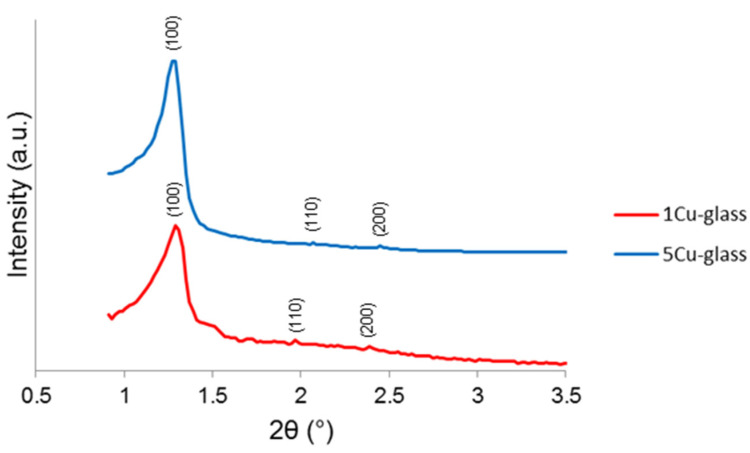
Small-angle XRPD patterns of copper-doped materials after calcination.

The small-angle XRPD patterns of both glasses (Figure 3) show three diffraction peaks that can be attributed to the (100), (110) and (200) reflections of a two-dimensional hexagonal *p6mm* lattice [23]. The d_100_ values were 6.7 and 6.8 nm 1Cu-glass and 5Cu-glass, corresponding to cell parameters of 7.7 and 7.8 nm (assuming a perfect two-dimensional hexagonal symmetry).

**Figure 4:**
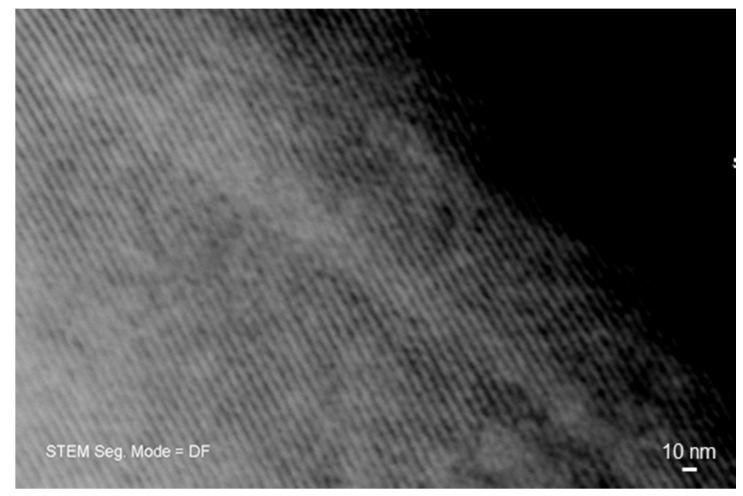
Scanning-transmission (STEM) image of 5Cu-glass recorded along the [100] direction, showing a parallel arrangement of nano-sized channels (mesopores).

The mesoporous nature of the materials was further confirmed by STEM investigation along the [100] direction (Figure 4), which allowed revealing an ordered arrangement of parallel one-dimensional nanopores (nano-channels). Rough measurements of pore dimeter yielded a value of around 5.7 nm.

**Figure 5:**
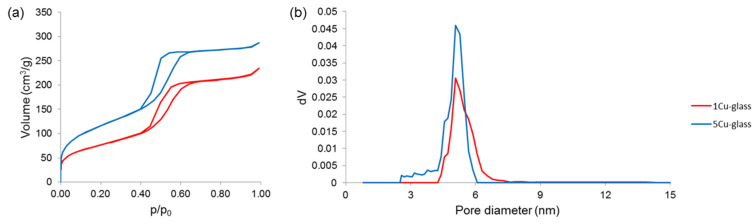
Nitrogen adsorption-desorption measurements performed on calcined glasses: (**a**) isotherms and (**b**) pore size distributions.

Nitrogen adsorption–desorption measurements (Figure 5) further confirmed the existence of uniform nanopores in the mesoscale range. Both glasses exhibited a type-IV isotherm, which is associated to pores with size between 2 and 50 nm (i.e., mesopores, according to the International Union for Pure and Applied Chemistry (IUPAC) definition) [29]. The shape of the hysteresis loop could provide information about the pore shape [30]: in both materials, the loop shape suggests the presence of cylindrical mesopores with hexagonal symmetry, which is typical of MCM-41 ordered mesoporous silica [31]. These results are in good agreement with the findings from small-angle XRPD (Figure 3). Quantification of textural features is summarized in Table 1; the value of pore size (5.1 nm) is close to that determined more roughly by STEM measurements (5.7 nm).

The data shown in Table 1 reveal that the pore volume and SSA of the mesoporous glasses decrease as the copper content increases. These results suggest that the incorporation of Cu^2+^ ions may have a negative effect on the precursor condensation, disrupting the ordered orientation of (SiO_4_)^4−^ units during the self-assembling reaction of the glass. Interestingly, although the total amount of modifiers (calcium and copper) is equal to 20 mol.% in all materials, the “disturbing” effect is higher when different types of modifiers are simultaneously introduced. This effect was also observed elsewhere when other modifiers (e.g., zinc or cerium) were added to the silicate network of mesoporous silicate glasses [32]. The mechanism behind this effect in mesoporous glasses is still to be elucidated, but a role could be played by the higher difference of modifier’s ionic radius as compared to silicon, which is the major forming element of the glass network (ionic radii: 0.210 nm for Si^4+^, 0.231 nm for Ca^2+^ and 0.140 nm for Cu^2+^, hence the absolute differences |∆_Si-Cu_| = 0.070 nm > |∆_Si-Ca_| = 0.021 nm).

The copper-depending trend of pore volume and SSA displayed in Table 1 is also consistent with that recently observed by Luo et al. [33] for Cu-doped nanofibrous mesoporous glass scaffolds. Unlike pore volume and SSA, the mean pore size is not apparently affected by the increasing content of copper in the glass composition.

SSA of all mesoporous glasses collected in Table 1 is significantly higher than that assessed for both melt-derived (less than 1 m^2^/g) and sol-gel silicate glasses produced without using a structure directing agent (few tens of m^2^/g) [34]; this is consistent with previous findings on several mesoporous glass types and compositions [35,36].

**Figure 6:**
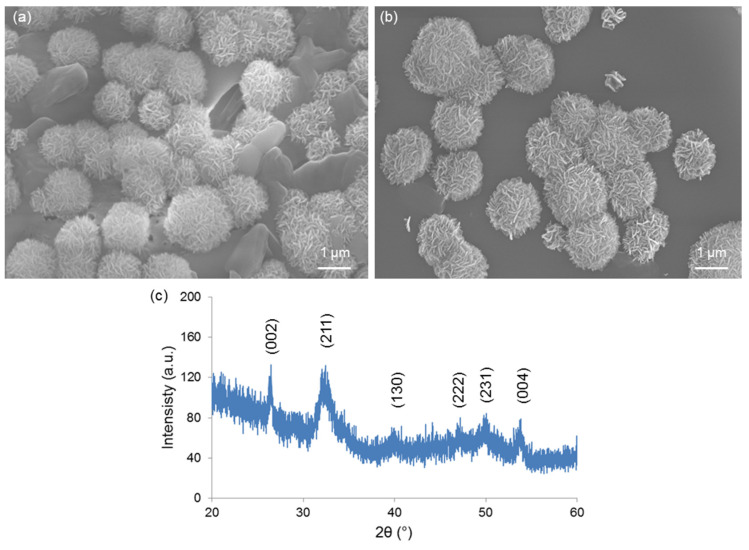
In vitro bioactivity tests: SEM micrographs showing the “cauliflower” calcium-phosphate agglomerates formed on (**a**) 1Cu-glass and (**b**) 5Cu-glass after immersion for 2 weeks in SBF; (**c**) XRD analysis on 5Cu-glass (2 weeks in SBF), which reveals the diffraction peaks of hydroxyapatite formed during the test.

Figure 6a,b reveals the formation of calcium phosphate globular agglomerates on the surface of both Cu-doped materials after immersion in SBF, thus demonstrating the apatite-forming ability of these glass compositions in vitro. The newly-formed phase exhibit a “cauliflower” structure formed by needle-like nano-sized crystals: this is the typical morphological “fingerprint” of the hydroxyapatite-like phases grown on the surface of bioactive glasses upon soaking in SBF. Semi-quantitative compositional assessment (EDS) on the agglomerates formed on 1Cu-glass and 5Cu-glass yielded Ca-to-P atomic ratios of 1.85 and 1.91, respectively. These values are higher than the Ca-to-P atomic ratio of stoichiometric hydroxyapatite (1.67), but can be justified considering the boundary effects due to the finite volume involved in compositional assessment by EDS. On the other hand, non-stoichiometric hydroxyapatite has been reported to commonly form on silicate glasses in SBF [37,38]. XRD analysis (Figure 6c) confirmed the formation of hydroxyapatite (PDF code: 01-086-0740) on the surface of samples during immersion in SBF.

At present, the biomaterials community assumes that the formation of a hydroxyapatite-like layer on the surface of a given material soaked in SBF is a proof of its bioactivity and, to some extent, of its bone bonding ability in vivo [26]. Being bioactive, the Cu-doped mesoporous materials produced in this work can be included in the versatile class of mesoporous bioactive glasses (MBGs), which have attracted great interest over the last few years for potential use in tissue engineering applications [39].

**Figure 7:**
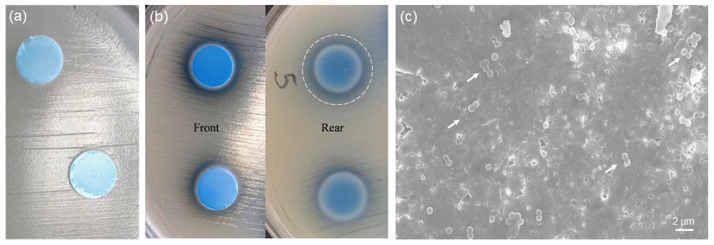
In vitro antibacterial experiments (24 h): inhibition halo tests on (**a**) 1Cu-glass and (**b**) 5Cu-glass; (**c**) SEM micrograph showing the surface of 1Cu-glass after the test (the white arrows highlight the few clusters of bacteria survived).

Previous studies showed that MBGs with high silica content (>80 mol.%) are highly versatile carriers for antibiotics but exhibit negligible [19] or no antibacterial effect (equivalent effect to that of plastic control [40]) in the short term if used alone. Therefore, incorporation of copper was thought as a valuable strategy to impart inherent antimicrobial extra-functionalities to these silicate biomaterials. The results of the antibacterial tests performed on 1Cu-glass and 5Cu-glass discs are shown in Figure 7. As shown in Figure 7a, 1Cu-glass composition is apparently unable to create an inhibitory halo for bacteria around the sample. On the contrary, an inhibitory halo can be clearly observed around the 5Cu-glass sample (region of total inhibition around 2 mm around the outer surface) (Figure 7b). These results suggest that the initial concentration of copper in the MBG composition is key in dictating the antibacterial behavior, in agreement with previous observations reported by other authors. However, we should take into account that antibacterial materials can exert their antiseptic effect via (i) release-killing mode, which is due to the release of antibacterial ions, and/or (ii) contact-killing mode, if bacteria come in direct contact with the biomaterial. Hence, the visual inspection of the surface of 1Cu-glass sample is important to clarify whether a contact-killing antibacterial effect can still be elicited. In this regard, the lack of bacteria on the surface of the sample brought into contact with the bacteria-inoculated plate during the Kirby-Bauer test can provide an evidence of contact-mode antibacterial capacity. Figure 7c shows a SEM image of the 1Cu-glass surface after the Kirby-Bauer test: although this samples showed no inhibition halo, the image clearly shows that only few clusters of *Staphylococcus aureus* survived after 24 h of incubation on this material. Furthermore, it is worth underlining that the antibacterial effect of copper ions was reported to be more significant against Gram-negative bacteria, such as *Escherichia Coli*, compared to Gram-negative strains such as *Staphylococcus Aureus* [19], which could partially justify the absence of a clear antibacterial halo around the sample doped with the lower amount of copper. In summary, these early results demonstrate that both copper-doped MBG compositions exhibit an antimicrobial effect against *Staphylococcus aureus* and motivate further investigation on these highly promising bioactive and antibacterial multifunctional biomaterials.

## 4. Conclusions

Copper-doped MBGs were obtained by a wet (sol-gel) route in which a non-ionic surfactant was incorporated as a mesopore template. After calcination, the glasses exhibited an ordered structure of mesopores arranged according to hexagonal symmetry. The presence of a mesoporous texture was the reason behind the apatite-forming property of these glasses despite the high content of silica (80 mol.%), as the large SSA (275–450 m^2^/g) was key to enhance the ion-exchange phenomena between glass and solution during immersion in SBF. The amount of copper in the MBG composition played a role in affecting both textural and functional properties: as copper increased, the SSA decreased but the antibacterial effect against *Staphylococcus aureus* was more significant. These preliminary observations show promise for the potential use of copper-doped MBGs in bone tissue engineering applications and motivate further investigation on these materials. 

## Figures and Tables

**Table 1:** Textural characteristics of calcined mesoporous glasses obtained by nitrogen adsorption-desorption porosimetry.

Sample	Pore Volume (cm^3^/g)	SSA (m^2^/g)	Mean Pore Size (nm)
0Cu-glass	0.265	450	5.0
1Cu-glass	0.232	432	5.1
5Cu-glass	0.165	275	5.1
